# Use of remote sensing to identify spatial risk factors for malaria in a region of declining transmission: a cross-sectional and longitudinal community survey

**DOI:** 10.1186/1475-2875-10-163

**Published:** 2011-06-10

**Authors:** William J Moss, Harry Hamapumbu, Tamaki Kobayashi, Timothy Shields, Aniset Kamanga, Julie Clennon, Sungano Mharakurwa, Philip E Thuma, Gregory Glass

**Affiliations:** 1 Department of Epidemiology, Bloomberg School of Public Health, Johns Hopkins University, Baltimore, Maryland, USA; 2 Harry Feinstone Department of Molecular Microbiology and Immunology, Bloomberg School of Public Health, Johns Hopkins University, Baltimore, Maryland, USA; 3 Macha Research Trust, Choma, Zambia; 4 Department of Biostatistics and Bioinformatics, Rollins School of Public Health, Emory University, Atlanta, Georgia, USA

## Abstract

**Background:**

The burden of malaria has decreased dramatically within the past several years in parts of sub-Saharan Africa. Further malaria control will require targeted control strategies based on evidence of risk. The objective of this study was to identify environmental risk factors for malaria transmission using remote sensing technologies to guide malaria control interventions in a region of declining burden of malaria.

**Methods:**

Satellite images were used to construct a sampling frame for the random selection of households enrolled in prospective longitudinal and cross-sectional surveys of malaria parasitaemia in Southern Province, Zambia. A digital elevation model (DEM) was derived from the Shuttle Radar Topography Mission version 3 DEM and used for landscape characterization, including landforms, elevation, aspect, slope, topographic wetness, topographic position index and hydrological models of stream networks.

**Results:**

A total of 768 individuals from 128 randomly selected households were enrolled over 21 months, from the end of the rainy season in April 2007 through December 2008. Of the 768 individuals tested, 117 (15.2%) were positive by malaria rapid diagnostic test (RDT). Individuals residing within 3.75 km of a third order stream were at increased risk of malaria. Households at elevations above the baseline elevation for the region were at decreasing risk of having RDT-positive residents. Households where new infections occurred were overlaid on a risk map of RDT positive households and incident infections were more likely to be located in high-risk areas derived from prevalence data. Based on the spatial risk map, targeting households in the top 80^th ^percentile of malaria risk would require malaria control interventions directed to only 24% of the households.

**Conclusions:**

Remote sensing technologies can be used to target malaria control interventions in a region of declining malaria transmission in southern Zambia, enabling a more efficient use of resources for malaria elimination.

## Background

The burden of malaria has decreased dramatically in parts of sub-Saharan Africa [1]. While some of this decline is attributable to intensified control efforts, particularly the use of more effective anti-malarial drugs and the scale-up of insecticide-treated nets (ITNs) and indoor residual spraying (IRS), the situation is more complex [2]. In several sites the decline in malaria preceded widespread introduction of control strategies. In Kilifi, Kenya, for example, the decline in paediatric hospitalizations for malaria began a year before the large-scale distribution of ITNs and two years before the availability of artemisinin-combination therapy (ACT) [3].

Declines in the burden of malaria have been observed in Zambia, coincident with the widespread implementation of malaria control strategies, including the use of ACT as the first-line treatment regimen, the distribution of long-lasting insecticidal nets (LLINs), and targeted IRS [4]. The overall prevalence of malaria parasitaemia in children younger than five years of age decreased 53% from a baseline prevalence of 22% between 2006 and 2008 [5]. In Choma District, southern Zambia, paediatric hospitalizations at Macha Hospital for malaria decreased from approximately 1,400 admissions per malaria season in 2000-2001 to fewer than 50 per year in each of the past three years. This decline began in 2004, shortly after the introduction of ACT, but before widespread distribution of LLINs.

The decline in the burden of malaria in parts of sub-Saharan Africa has led to interest in malaria elimination and eradication [6]. One strategy for regional elimination is a step-wise approach starting with countries at the margins of endemic transmission [7], including the southern margins of malaria transmission in Africa [8]. Within the countries of southern Africa there is a range of *Plasmodium falciparum *parasite rates, corresponding to different levels of endemicity and phases of control [9]. Understanding the local epidemiology of malaria in southern African is thus critical to assessing the feasibility of regional malaria elimination.

Targeting interventions to hotspots of malaria transmission results in more efficient and cost-effective accelerated malaria control efforts [10] but requires identification of individual, household, and environmental correlates of transmission. Remote sensing technologies were applied to determine if readily generated environmental data are of sufficient detail and validity to identify heterogeneity in spatial risk factors for malaria transmission in southern Zambia, where the burden of malaria has decreased and elimination may be feasible.

## Methods

### Study population

**Figure 1:**
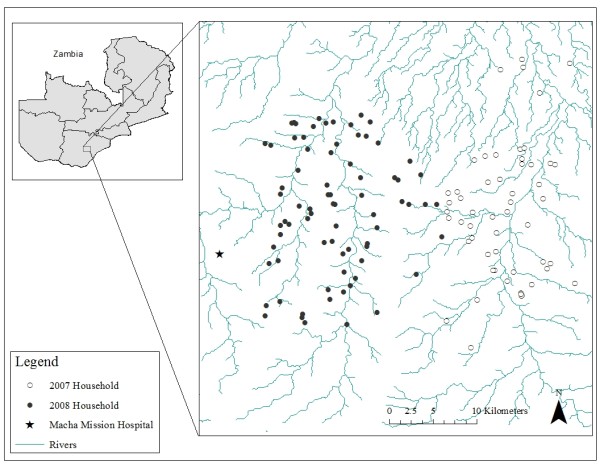
**Map of the study sites in Choma District, Southern Province, Zambia**.

Satellite images were used to construct a sampling frame for the random selection of households enrolled in prospective longitudinal and cross-sectional surveys of malaria parasitaemia in the catchment area of Macha Hospital in Southern Province, Zambia (Figure 1). Macha Hospital is approximately 70 km from the nearest town of Choma and the catchment area is populated by traditional villagers living in small, scattered homesteads. *Anopheles arabiensis *is the primary vector responsible for malaria transmission, which peaks during the rainy season from December through April [11]. The sampling frame for the random selection of households was constructed from a Quickbird™ satellite image obtained from DigitalGlobe Services, Inc. (Denver, Colorado). The image was imported into ArcGIS 9.2 (Environmental Systems Research Institute [ESRI], Redlands, California) and locations of households were identified and enumerated manually. Structures of appropriate size and shape were identified as potential residences, and consisted of one or more domestic structures where members of a family resided. Smaller structures, such as kraals, and larger structures, such as schools, were excluded. Selected households were allocated to one of two study cohorts: longitudinal and cross-sectional. Households in the longitudinal cohort were surveyed repeatedly approximately every two months and households in the cross-sectional cohort were surveyed once. Cross-sectional and longitudinal household surveys were conducted approximately every other month (during alternate months) from April 2007 through December 2007 in the first study area and from February 2008 through December 2008 in the second study area. Data from all cross-sectional households and the first longitudinal household visit were used to develop the spatial risk model. Model validation was conducted with the full longitudinal dataset. The study was approved the Johns Hopkins Bloomberg School of Public Health Institutional Review Board and the University of Zambia Research Ethics Committee.

A field team was provided with images and coordinates of the randomly selected households. After obtaining permission from the local chief and head of household, and individual written informed consent, a questionnaire was administered to each participant residing within the household and a blood sample was collected by finger prick. Rapid diagnostic tests (RDT; ICT Diagnostics, Cape Town, South Africa) were used to detect *P. falciparum *histidine-rich protein 2. This RDT was shown to detect 82% of test samples with wild-type *P. falciparum *at a concentration of 200 parasites/μL 98% of test samples with a concentration of 2,000 parasites/μL, with false positives in 0.6% of clean negative samples [12]. Individuals who were RDT positive were offered treatment with artemether-lumefantrine (Coartem^®^) by trained medical personnel. Households in which at least one individual tested positive by RDT were classified as a positive household. Positive and negative households were plotted as a data layer in ArcGIS 9.2.

### Landscape characterization

A digital elevation model (DEM) for the area with 1 m horizontal resolution was derived from the Shuttle Radar Topography Mission (SRTM) version 3 DEM with 90 m pixels. Elevation values correspond to the reflective surface on the earth and represent soil surface, vegetation or man-made structures. The SRTM imagery was collected during a 2001 space shuttle mission using a multi-frequency, multi-polarization radar system. Each pixel represented a 30 m average elevation around each pixel's center. The relative horizontal accuracy was ±15 m (90% circular error) with a relative vertical accuracy of ±6 m (90% vertical error).

### Topographic wetness

The digital elevation model was processed in Imagine 9.1 (Earth Resource Data Analysis System [ERDAS], Norcross, Georgia) and imported into ArcGIS 9.2. Imagery and point locations were geo-referenced to UTM zone 35S, WGS 1984. The ArcGIS extension Terrain Analysis Using Digital Elevation Models extension [13] was used to model water flow and calculate an index of topographic wetness (ITW). Data were smoothed to fill in isolated elevation pits or spikes typically representing errors or areas of internal drainage that interrupt estimates of water flow. Slope and flow directions were determined using the multiple direction algorithm (MDA) [14] and the flat area flow direction methods [15]. The MDA method used the steepest slope of triangular facets, allowing water to flow in any direction. The ITW is an indicator of potential moisture, assuming surface homogeneity for soil and vegetation, and is calculated using the ratio of upslope contributing area and local slope (the tangent of slope = tanβ). A pan-sharpened Quickbird imagery scene (DigitalGlobal, Longmont, Colorado) (2.5 m mulitspectral) collected on June 12, 2007 was used to evaluate the hydrological model. Drainages were categorized according to the Strahler stream order classification into first through fifth order streams [16,17], such that, for example, a second order stream is formed when two first order streams join.

### Topographic position index

The topographic position index (TPI) was generated in ArcView 3.3 (ESRI, Redlands, California) with an extension by Jenness [18]. The TPI classifies the landscape by slope position (low, middle, high) and landform type (plain, valley, ridge), and represents the difference between the elevation at a point and the elevations of neighbourhood cells. TPI values near zero are typical of flat or mid-slope locations. High values signify areas, such as hill tops and ridges, while low values are indicative of valley floors. Because the TPI is scale dependent, local (500 m) and area-wide (2 km) scales were considered. The 500 m neighbourhood detected local valleys and hills while the 2 km neighbourhood identified larger scale features such a large U-shaped valleys, gently sloped hills and tops of plateaus.

The magnitude of the TPI and the area's slope were used to classify the slope position according to Weiss [19], based on the TPI score standard deviations and slope values. Slope position classes were valley, lower slope, flat slope, middle slope, upper slope and ridge. Ten landform classes (deep streams, shallow valleys and mid-slope drainage pathways, upland drainage areas, U-shaped valleys, plains, open slopes, upper slopes and mesas, local ridges and hills in large valleys, mid-slope of ridges and small hills in plains, and high ridges) were generated by comparing standardized TPI values (standardized TPI = [TPI - TPI mean]/[TPI standard deviation]) for TPI values at 500 m (TPI_500_), 2 km (TPI_2,000_) and slope [19].

### Statistical analyses

Incident malaria infections were estimated using data from the longitudinal survey. An incident infection was defined as an individual with a positive RDT after a prior negative RDT, or an individual with two consecutive positive RDTs more than 30 days apart. The month of infection was taken as the mid-point between a negative and positive RDT test, or the midpoint between one month after the first positive RDT and the time of the subsequent positive RDT. Logistic regression was used to identify environmental conditions associated with the odds of a household having an individual with a positive RDT. Distances of the surveyed households from the five stream orders were aggregated into quartiles. Household elevation above the study area baseline (1,009 m) in 10 m increments and slope were included in the model as continuous variables. Aspect was coded so that households with an eastern or south-eastern exposure were coded as 1, those with western and northwestern exposure were coded -1 and all others as 0. Analyses were performed using Statistix version 8.0 (Analytical Software, Tallahassee Florida). The spatial structure of residual errors was examined using Global Moran's I to determine if there was a systematic departure from the assumption that errors were spatially independent (i.e. spatially autocorrelated), which would overinflate the significance of the environmental risk factors estimated by logistic regression.

## Results

### Characteristics of the study population

**Figure 2:**
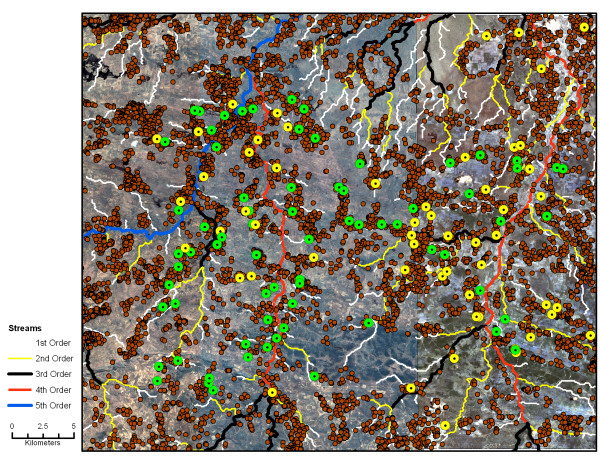
**Spatial distribution of households with and without RDT positive individuals**. Brown circles indicate all households in the study area. Yellow circles indicate sampled households in which at least one resident was RDT positive at the study visit. Green circles indicate sampled households in which no resident was RDT positive at the study visit. First through fifth order streams are indicated.

There were 8,751 households identified from the Quickbird satellite image in the study area (Figure 2). A total of 768 individuals from 128 randomly selected households were enrolled over 21 months (Figure 2), from the end of the rainy season in April 2007 through December 2008. The 128 households represented 1.5% of the 8,751 households documented in the 1,650 km^2 ^study area. The study area surveyed from April to December 2007 was east of the study area surveyed from January to December 2008 (Figure 1). Thirty-five households were enrolled in the longitudinal survey and were visited more than once. Initial RDT results from the 270 individuals residing within these 35 households were used to characterize the prevalence of malaria in these households. These data were combined with the 498 individuals in 93 households enrolled in the cross-sectional survey.

The median age of study participants was 12.8 years (interquartile range [IQR]:5.2, 31.8) in 2007 and 14.3 years (IQR: 6.4, 34.3) in 2008. Of the 768 individuals tested, 117 (15.2%) were positive by RDT. These individuals resided in 56 (43.8% of 117) households, with 36 (69.2% of 56) households enrolled in 2007 and 20 (26.3% of 56) households enrolled in 2008. The prevalence of RDT positive individuals in the 2007 longitudinal survey was 25.5% compared with 27.2% in the cross-sectional survey. The prevalence of RDT positive individuals was 4.3% in the longitudinal survey in 2008 (west of the 2007 study area), compared with 9.8% in the cross-sectional survey.

### Landforms and aspect of sampled households were representative of households in the study area

Landforms occupied by the surveyed households were similar to those not surveyed in the study area. Surveyed (n = 128) and non-surveyed (n = 8,623) households were found on plains (85% vs. 75%, respectively), mid-slope drainage pathways (6% vs. 7%), mesas (4% vs. 6%), ridges (3% vs. 5%), canyons (2% vs. 2%), U shaped valleys (0% vs. 5%) and open slopes (0% vs. 0.002%). Households in U-shaped valleys were not represented in the surveyed households, as were two landforms not present in the study area, open slopes and headwaters.

The aspect of the land described by the compass direction of the steepest slope for an individual pixel was used to determine the direction the sloping land faced (flat surfaces were assessed separately). Generally, western and northwestern facing slopes are warmer and drier than comparable eastern and south-eastern facing slopes in the southern hemisphere. The distribution of slope directions for all households within the study area was proportional to the slope directions for land in the study region, indicating residents did not preferentially select a particular aspect when constructing households (see Additional File 1). Land aspects of the surveyed households matched the distribution of the non-surveyed households, with the exception of slight under sampling of households on northwestern facing slopes and a slight excess of households facing north-east (see Additional File 1).

### Lower elevation was associated with increased risk of malaria

The elevation of households in the study area ranged from 1,009 to 1,268 m above sea level. Households with RDT positive individuals were significantly lower in elevation on average (elevation mean + standard deviation: 1,079.1 + 28.92 m; range 1,014 to 1,143 m) than households with only RDT negative individuals (1,105.6 + 46.21 m; range 1,040 to 1,247 m) (P = 0.0001).

### Lesser slope was associated with increased risk of malaria

Households with RDT positive individuals were approximately 50% more likely than RDT negative households to occupy eastern and south-eastern facing slopes (see Additional File 1). Conversely, RDT negative households were twice as likely to occupy north-western and western-facing slopes compared to RDT positive households. The slope of the land, as measured in degrees, characterizes the rate at which the elevation of the local area changes. The study area was generally flat, with the average slope surrounding households 0.026° ± 0.019°. On average, RDT positive households were on flatter ground (slope of 0.024° ± 0.014°) than RDT negative households (slope of 0.031° ± 0.023°; p = 0.04).

### Proximity to third order streams was associated with increased risk of malaria

The risk of malaria associated with living increasingly closer to each of the five stream orders was assessed using logistic regression, with the furthest distance used as the reference category. Households with at least one RDT positive individual were compared with households in which no individuals were RDT positive. Only distances from third and fifth order streams were associated with the odds of a household having an RDT positive individual. The risk decreased the closer a household was to the only fifth order stream in the region. Households within 4.5 km of a fifth order stream were 2.6 (95% CI 1.1-6.2) times less likely to be positive than households greater than 20.6 km from a fifth order stream. In contrast, living in proximity to a third order stream increased the risk of RDT positivity. Households within 1.98 km of a third order stream were 2.8 (95% CI 1.2-6.9) times more likely to have an RDT positive resident than households situated more than 6.0 km from a third order stream.

The multivariable logistic model identified the nearest quartile of distance to third order streams, and between the first and median quartiles of distances to third order streams, as significant predictors of the odds of a household having an RDT positive individual (Table 1). There was an increased risk of malaria for persons living within 3.75 km of a third order stream. Households at elevations above the baseline elevation for the region (1009 m) were at decreasing risk of having an RDT positive individual. For every 10 m rise in elevation the risk decreased by approximately 13%. No other environmental characteristics were significantly associated with the odds of a household having an RDT positive individual in the multivariable analyses. The residual errors did not show significant spatial autocorrelation (I = -0.07), so alternative model structures were not incorporated. Generalized linear mixed models were evaluated but they did not improve the fit of the model and were not considered further.

**Table 1:** Spatial risk factors for malaria

	Number of households	% Positive households	Unadjusted OR (95% CI)	***P***	Adjusted OR (95% CI)	***P***
**Third order streams**						
<1.98 km	31	18	2.85 (1.18, 6.9)	0.020	3.93 (1.55, 9.93)	0.0008
1.98-3.74 km	33	16	1.80 (0.76, 4.2)	0.179	3.47 (1.20, 10.05)	0.004
3.74-5.95 km	32	13	1.08 (0.44, 2.7)	0.862	1.38 (0.52, 3.70)	0.194
> 5.95 (ref)	32	13	1.00			
**Fifth order streams**						
<4.55 km	32	7	0.39 (0.16, 0.91)	0.030		
4.55-11.92 km	32	8	0.22 (0.09, 0.58)	0.002		
11.92-20.57 km	32	17	0.44 (0.18, 1.09)	0.078		
> 20.57 (ref)	32	23	1.00			
**Aspect**						
Eastern or south-eastern	32	18	2.06 (0.91, 4.70)			
Western or north-western	20	5	0.43 (0.14, 1.32)			
	**Negative households**	**Positive households**				
**Elevation above baseline (1009 m)**						
10 m increments	1105.6 ± 46.2 m	1097 ± 48.2 m	0.95 (0.91, 0.98)	0.006	0.87 (0.81, 0.95)	0.0001
**Slope**	0.03 ± 0.023	0.02 ± 0.014				

### Spatial risk map for malaria

**Figure 3:**
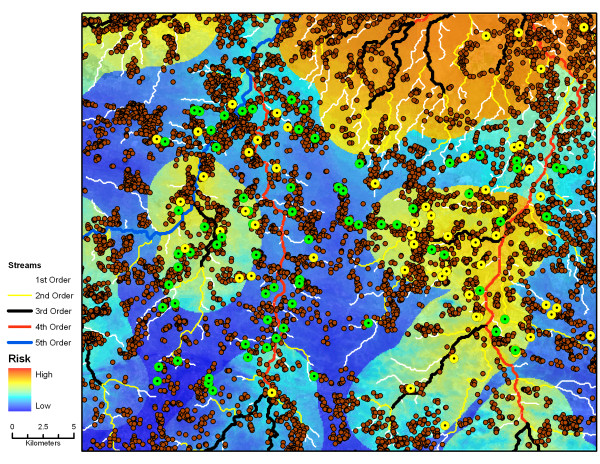
**Malaria risk map**. Malaria risk map generated using households with prevalent RDT-positive malaria cases, showing all households in the study area and first through fifth order streams. Yellow and orange shading indicates regions of high risk and blue shading indicates regions of low risk.

A spatial risk map for the study area was generated based on the regression analyses (Figure 3). Areas of higher risk for malaria increased in the north of the study region and in proximity to third order streams. However, there was a large region of low risk between the northern region and the two fourth order streams running south.

### Validation of the risk map using incidence cases

An estimate of incident malaria cases from the longitudinal surveys was used to evaluate the predictive ability of the risk map based on prevalence data and to locate areas where new malaria cases were identified.

A total of 102 individuals from 17 households were enrolled in the longitudinal survey in 2007. A single RDT was performed on 27 individuals (three of whom were RDT positive) and they were excluded from analysis. The remaining 75 individuals contributed 410 person-months at risk, the period at risk being determined by the interval in weeks between negative tests and allowing for a 30-day period of no risk after treatment of an individual with a positive RDT. There were 37 incident malaria infections in 2007 (20 of which were in individuals with a positive RDT after a previous negative test and 17 were consecutive positive RDTs in the same individual more than 30 days apart), resulting in 9.02 events/100 person-months of observation. Nine incident cases were identified in April, 15 in May, 10 in June, two in August and one in September.

A total of 117 individuals from 19 households were enrolled in the longitudinal survey in 2008. A single RDT was performed on 17 individuals and they were excluded from analysis (none of whom were RDT positive). The remaining 100 individuals contributed 771 person-months at risk. There were four events in 2008 (three of which were in individuals with a positive RDT after a previous negative test and one of which was due to consecutive positive RDTs in the same individual more than 30 days apart), resulting in 0.52 events/100 person-months of observation in 2008. One incident case occurred in March, two in April and one in May.

**Figure 4:**
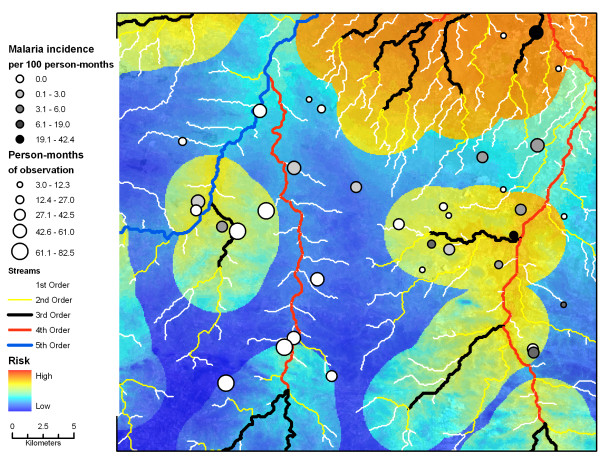
**Incident malaria cases and the malaria risk map based on prevalent cases**. Households with and without incidence malaria cases detected by RDT overlaid on the malaria risk map and first through fifth order streams. The size of the circle represents the number of person-years of observation within each study household.

**Figure 5:**
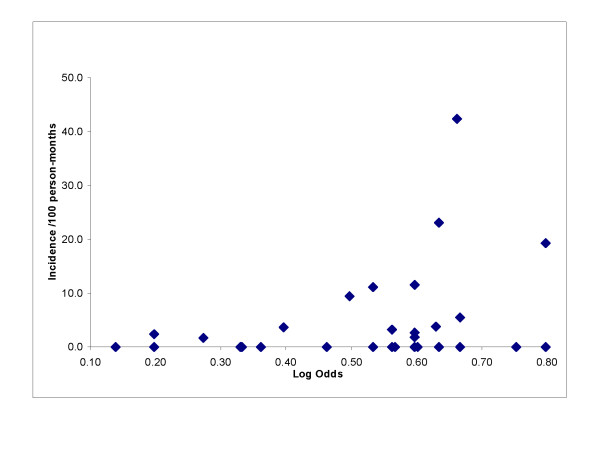
**Household incidence of malaria compared with log odds of residing in a high risk area**.

In total, there were 1,181 person-months of risk for 175 people during 21 calendar months in 2007 and 2008. There were 41 RDT events or 3.47/100 per person-months of observation. Households where new infections occurred were overlaid on the risk map of RDT positive households (Figure 4). Incident infections were more likely to be located in high-risk areas based on the prevalence data. The incidence of an RDT positive individual within a household showed a rapid rise as the value of the logistic function exceeded 0.52 (Figure 5). In households where the function exceeded 0.60, household incidence of an RDT positive individual averaged 10%/month compared with 1.9%/month for households with lower risk (Figure 5).

### Using the spatial risk map to target malaria control interventions

To explore the consequences of identifying households for targeted interventions based on the risk map, the proportion of households above different thresholds in predicted risk of having an RDT positive individual was determined and these estimates were extrapolated to all the households in the region. To identify 50%, 80% and 90% of the households with RDT positive individuals, logit estimates of p > 0.53, 0.31 and 0.26, respectively, were used from the empirical distribution of predicted values. Among the surveyed households, this resulted in fewer households with RDT positive individuals being excluded (27, 11 and 5 households with at least one RDT positive individual being missed, respectively). As a measure of specificity, these thresholds also included 43, 21, and 17 households with no RDT positive individuals, so that 62%, 66% and 77% of households below these thresholds would have no RDT positive individuals.

In addition to the presence of individuals with RDT positive results in the households, there also was a substantial difference in the frequency of infection as measured by the proportion of tests performed that gave positive results. For example, when the threshold was selected to capture 90% of the households with any RDT positive results, only five of the 338 (1.5%) RDT tests performed in households below the threshold were positive. By comparison, in households above the threshold there were 40 households with at least one RDT positive individual and there was a substantially higher proportion of positive RDTs among individuals residing in these households (114/579 = 19.7% tests).

To target malaria control interventions to all households at or above the 90^th ^percentile of predicted risk would require that 38.8% (3,398) of the 8,751 households in the region receive the intervention. Households in the top 80^th ^percentile would require targeting of malaria control interventions to 23.6% (2,067) of the households, and the top 50^th ^percentile required targeting interventions to 20.3% (1,775) of the households.

## Discussion

Remote sensing enabled identification of environmental risk factors for *P. falciparum *parasitaemia in a region of declining malaria transmission in southern Zambia and the generation of a spatial risk map predictive of incident malaria cases. Proximity to third order streams and low altitude were environmental characteristics associated with households in which a resident had parasitaemia, presumably because of proximity to anopheline breeding sites in these ecological settings. A strength of this methodology is that parasite prevalence was measured in symptomatic and asymptomatic individuals residing in randomly selected households using satellite images and was not subject to biases inherent in passive detection at health care facilities or limited to symptomatic individuals. Furthermore, the landforms of surveyed households were representative of all households in the study area. This study is one of the first to identify environmental risk factors for malaria in a region of declining malaria transmission and accelerated control efforts. Using data readily generated with remote sensing technologies, this approach can be used to guide targeted malaria control interventions to further reduce malaria transmission.

Remote sensing has been used to identify risk factors for malaria in regions of high malaria transmission [20]. Commonly identified risk factors include land elevation [21] and proximity to vector breeding sites. In a region of high malaria endemicity in Ghana, small-scale heterogeneity in risk was identified between villages, with increased risk in those households closer to the forest fringe [22]. Prior studies have identified proximity to bodies of water as a risk factor for malaria, although none identified a specific stream order. In a region of low malaria endemicity in northern Tanzania, living close to the river was an independent predictor for malaria infection based on passive case detection at a health care facility, and the authors suggest that interventions should be targeted to households close to the river [23]. Hydrologic modelling was used to assess the risk of malaria in the highlands of western Kenya [24]. Topography-derived wetness indices were significantly associated with household-level malaria incidence, independent of elevation. Specifically, households with cases of malaria were located 280 m closer to regions with high wetness indices than control households. However, in a highly malaria endemic area in western Côte d'Ivoire, proximity to a river was not significantly associated with the risk of malaria after adjusting for spatial correlation [25].

A malaria risk map was generated based upon the environmental risk factors that allowed us to extrapolate malaria risk throughout the study area. This map was based on parasite prevalence data from randomly selected households and validated with incidence data, confirming the biological significance of the identified risk factors. Other, potentially more biased, sampling methods have been used to generate malaria risk maps. For example, local clustering was identified and risk maps generated from malaria morbidity data in East Shoa, Ethiopia and digital elevation models [26], and risk models have been generated on smaller scales [27]. Generating risk maps from modelling approaches such as logistic regression makes strong assumptions about the form of the residual variation, especially that the errors are independently distributed rather than retaining spatial structure. Thus, testing for spatial structure in the residual variation is needed to ensure that the significance of the point estimates of the environmental risk factors is not overestimated, and that at the scale of the analysis remaining, undetected co-factors have not been ignored.

## Conclusions

For national planning of malaria control strategies, large-scale maps are needed. Validated, remote sensing technologies allow generation of large-scale risk maps for targeting of malaria control interventions. Using readily available ecological data and remote sensing technologies, a malaria risk map was generated for targeted interventions in a region of decreasing malaria transmission in southern Zambia. This analysis suggests that increasing the targeted area from 50% to 80% of high-risk communities requires reaching a much smaller proportion of households than increasing the target area from 80% to 90% of high-risk households. Although different settings may have a different set of predictors, such information can guide allocation of limited resources to achieve further malaria control in regions of declining malaria transmission.

## Competing interests

The authors declare that they have no competing interests.

## Authors' contributions

WJM conceived of the study, participated in its design and coordination, and drafted the manuscript. HH participated in the design and coordination of field aspects of the study. TK participated in the coordination of the study and preparation of the manuscript. AK participated in the coordination of the study. JC carried out statistical analyses and participated in the preparation of the manuscript. SM participated in the design and coordination of the study. PET participated in the design and coordination of the study. GG conceived of the study, participated in its design, coordination and statistical analysis and drafted the manuscript. All authors read and approved the final manuscript.

### Funding

This work was supported by the Johns Hopkins Malaria Research Institute and the Bloomberg Family Foundation.

## Supplementary Material

Additional file 1 **The percentage of households situated in different aspects of the land, by RDT positive and negative households, unsurveyed households and the total landscape**. Click here for file
