# Supplementary material for: Use of remote sensing to identify spatial risk factors for malaria in a region of declining transmission: a cross-sectional and longitudinal community survey
Source: Malar J. 2011 Jun 10;10:163. doi: 10.1186/1475-2875-10-163 (PMC3123248; doi:10.1186/1475-2875-10-163)
Supplement: Additional file 1 — The percentage of households situated in different aspects of the land, by RDT positive and negative households, unsurveyed households and the total landscape. [file 1475-2875-10-163-S1.DOC]

**Additional Files**

**Additional File**: The percentage of households situated in different aspects of the land, by RDT positive and negative households, unsurveyed households and the total landscape. Spatial resolution was 90 m. East and southeast facing aspects were more likely to have positive households, while northwest and west facing aspects were more likely to have negative households.

|  | **East** | **Northeast** | **North** | **Northwest** | **West** | **Southwest** | **South** | **Southeast** | **Flat** |
| --- | --- | --- | --- | --- | --- | --- | --- | --- | --- |
| **Positive households^1^** | 19.6 | 21.4 | 21.4 | 3.6 | 5.4 | 7.1 | 8.9 | 12.5 | 0 |
| **Negative households** | 11.1 | 23.6 | 22.2 | 8.3 | 12.6 | 5.6 | 8.3 | 8.3 | 0 |
| **Unsurveyed households** | 13.2 | 17.4 | 17.1 | 13.4 | 9.5 | 7.6 | 9.8 | 11.7 | 0.3 |
| **Landscape** | 13.0 | 18.1 | 18.7 | 13.4 | 9.6 | 8.0 | 8.6 | 10.3 | 0.4 |

^1^Positive households had at least one individual who was RDT positive.
